# The performance of metabolomics-based prediction scores for mortality in older patients with solid tumors

**DOI:** 10.1007/s11357-024-01261-6

**Published:** 2024-07-04

**Authors:** Yara van Holstein, Simon P. Mooijaart, Mathijs van Oevelen, Floor J. van Deudekom, Dina Vojinovic, Daniele Bizzarri, Erik B. van den Akker, Raymond Noordam, Joris Deelen, Diana van Heemst, Nienke A. de Glas, Cynthia Holterhues, Geert Labots, Frederiek van den Bos, Marian Beekman, P. Eline Slagboom, Barbara C. van Munster, Johanneke E. A. Portielje, Stella Trompet

**Affiliations:** 1 Department of Internal Medicine, Section of Gerontology and Geriatrics, Leiden University Medical Center, PO box 9600, 2300 RC Leiden, The Netherlands; 2 LUMC Center for Medicine for Older People, Leiden University Medical Center, Leiden, The Netherlands; 3 Department of Internal Medicine, Section of Nephrology, Leiden University Medical Center, Leiden, The Netherlands; 4 Department of Geriatric Medicine, OLVG Hospitals Amsterdam, Amsterdam, The Netherlands; 5 Department of Biomedical Data Sciences, Section of Molecular Epidemiology, Leiden University Medical Center, Leiden, The Netherlands; 6 Department of Epidemiology, Erasmus Medical Center, University Medical Centre, Rotterdam, The Netherlands; 7 Delft Bioinformatics Lab, Delft University of Technology, Delft, The Netherlands; 8 Max Planck Institute for Biology of Ageing, Cologne, Germany; 9 Cologne Excellence Cluster On Cellular Stress Responses in Ageing-Associated Diseases (CECAD), University of Cologne, Cologne, Germany; 10 Department of Medical Oncology, Leiden University Medical Center, Leiden, The Netherlands; 11 Department of Internal Medicine, Haga Hospital, The Hague, The Netherlands; 12 Department of Internal Medicine, University Medical Center Groningen, Groningen, The Netherlands

**Keywords:** Geriatric oncology, Geriatric assessment, Metabolomics, Biomarkers, Mortality

## Abstract

Prognostic information is needed to balance benefits and risks of cancer treatment in older patients. Metabolomics-based scores were previously developed to predict 5- and 10-year mortality (MetaboHealth) and biological age (MetaboAge). This study aims to investigate the association of MetaboHealth and MetaboAge with 1-year mortality in older patients with solid tumors, and to study their predictive value for mortality in addition to established clinical predictors. This prospective cohort study included patients aged ≥ 70 years with a solid malignant tumor, who underwent blood sampling and a geriatric assessment before treatment initiation. The outcome was all-cause 1-year mortality. Of the 192 patients, the median age was 77 years. With each SD increase of MetaboHealth, patients had a 2.32 times increased risk of mortality (HR 2.32, 95% CI 1.59–3.39). With each year increase in MetaboAge, there was a 4% increased risk of mortality (HR 1.04, 1.01–1.07). MetaboHealth and MetaboAge showed an AUC of 0.66 (0.56–0.75) and 0.60 (0.51–0.68) for mortality prediction accuracy, respectively. The AUC of a predictive model containing age, primary tumor site, distant metastasis, comorbidity, and malnutrition was 0.76 (0.68–0.83). Addition of MetaboHealth increased AUC to 0.80 (0.74–0.87) (*p* = 0.09) and AUC did not change with MetaboAge (0.76 (0.69–0.83) (*p* = 0.89)). Higher MetaboHealth and MetaboAge scores were associated with 1-year mortality. The addition of MetaboHealth to established clinical predictors only marginally improved mortality prediction in this cohort with various types of tumors. MetaboHealth may potentially improve identification of older patients vulnerable for adverse events, but numbers were too small for definitive conclusions. The TENT study is retrospectively registered at the Netherlands Trial Register (NTR), trial number NL8107. Date of registration: 22–10-2019.

**Supplementary Information:**

The online version contains supplementary material available at 10.1007/s11357-024-01261-6.

## Introduction

Older patients with solid tumors are a heterogeneous group and exhibit individual differences in physiological and functional characteristics, life expectancy, and treatment tolerance [1, 2]. It is important to identify patients with a poor prognosis to better balance the benefits and harms of cancer treatment in older patients. A geriatric assessment identifies geriatric deficits that predict treatment outcomes and thereby provides prognostic information in older patients with various types of cancer [3]. Blood biomarkers that provide additional prognostic information or improve prediction of treatment outcomes can potentially add value to known prognostic factors such as tumor characteristics and geriatric deficits.

Metabolomics-based biomarkers are of increasing interest in cancer and aging research. Metabolomics is the measurement of numerous molecules involved in metabolic processes and can be of additional value in cancer diagnosis and prognosis [4, 5]. A recent study showed that metabolomic profiles derived from a high-throughput proton nuclear magnetic resonance metabolomic platform were associated with cancer incident event rates in the UK Biobank cohort [6]. Previous studies in patients with metastatic breast cancer [7] and colorectal cancer [8] identified single metabolites or pathways that predicted overall survival. In patients with head and neck cancer [9] and lung cancer [10], metabolites were associated with treatment toxicity. In patients with cancer, it is also possible that metabolites change due to malnutrition and cachexia [11].

In aging research, blood metabolomics-based scores were constructed to provide generic indicators of overall health and vulnerability. These were either constructed to predict overall mortality [12] or to estimate an individuals’ biological age [13], and thereby mortality, in large population-based cohorts. Deelen et al. developed the MetaboHealth score for prediction of 5- and 10-year mortality [12]. Van den Akker et al. constructed the metabolomics-based age predictor MetaboAge [13], a calculation of the metabolomics age based on the relation of metabolites with chronological age. A positive difference between chronological and metabolomic age (ΔMetaboAge) was associated with a 25–42% increased risk of 10-year mortality depending on the studied cohort [13]. Next, the MetaboHealth score predicted frailty in a population based study [14] and mortality in older patients (mean age 82 years) with a femoral fracture [15]. As yet it is unclear whether these generic metabolite scores correlate to cancer mortality in geriatric patients. In addition, although MetaboHealth and MetaboAge can predict long-term mortality in large population-based cohorts, their performance on outcomes on the shorter term is unknown.

Therefore, the aims of the present study are to investigate the association of MetaboHealth and MetaboAge with baseline geriatric deficits and 1-year mortality in older patients with solid tumors, and to study their predictive value for mortality in addition to established clinical predictors.

## Methods

### Study design and setting

The current study is a substudy of the Triage of Elderly Needing Treatment (TENT) study, an ongoing Dutch prospective multicenter study that is embedded in a routine clinical care pathway. In the TENT study, patients aged ≥ 70 years who are candidate for intensive cancer treatment, such as (chemo-)radiotherapy, immune therapy, or major surgery, undergo a geriatric assessment before treatment initiation at a oncogeriatric care pathway. In routine care, interventions or pretreatment optimization were recommended according to existing guidelines when geriatric deficits were present [16]. Patients are followed until 1 year after treatment initiation or death. The TENT study was approved by the Medical Ethics Committee (ID number NL53575.058.15) of the Leiden University Medical Center (LUMC). All patients provided written informed consent. Details on the design and rationale of the oncogeriatric care pathway and TENT study were extensively described elsewhere [17].

### Patients

Eligible patients for this substudy were participants of the TENT study with a solid malignant tumor who underwent blood sampling and were included between August 2016 and October 2020 at LUMC (Leiden, the Netherlands) or Haga Hospital (The Hague, the Netherlands).

### Data collection

#### Patient characteristics

The following data were collected from digital patient files: age, sex, body mass index, smoking status and history, WHO performance score, primary tumor site, presence of distant metastasis, curative or palliative treatment intent, and treatment plan.

#### Geriatric assessment

Geriatric assessment data were collected according to the four geriatric domains: the somatic, psychological, functional, and social domains. Table 1 summarizes the tests used for the assessment of the four domains and their cutoff values. A domain had deficits if at least one individual test score was abnormal. Patients who had deficits in at least two domains were classified as frail. This definition of frailty was used in our previous work [18, 19] and showed a strong correlation with the 10-Item Frailty Index Based on a Comprehensive Geriatric Assessment [20] in the study by Baltussen et al. [19]. Data on geriatric assessment were extracted from digital patient files or Case Report Forms.

**Table 1:** Geriatric assessment

Geriatric assessment		Test	Range	Cutoff value
Somatic domain	Comorbidity	CCI [27]	0 to 33 points	≥ 1 points No points assigned to the “solid tumor” category if no other tumors were present
	Polypharmacy	N/A	N/A	Number of medications ≥ 5
	Malnutrition	MNA-SF® [28]	0 to 14 points	≤ 11 points
Psychological domain	History of delirium	N/A	N/A	Yes
	Dementia diagnosis	N/A	N/A	Yes
	Cognitive impairment	6CIT [29]	0 to 28 points	> 7 points
Functional domain	Fall incident	N/A	N/A	≥ 1 in past 6 months
	Institutionalization	N/A	N/A	Yes
	ADL dependency	Katz ADL [30]	0 to 6 points	≥ 2 points
	IADL dependency	Lawton IADL [31]	Men: 0 to 5 points Women: 0 to 8 points	Men: ≤ 4 points Women: ≤ 7 points
Social domain	Living situation	N/A	N/A	Living alone

*Abbreviation: 6CIT* Six-item Cognitive Impairment Test, *ADL* activities of daily l living, *CCI* Charlson Comorbidity Index, *IADL *instrumental activities of daily living, *MNA-SF*® Mini Nutritional Assessment Short Form, *N/A* not applicable

Patients who had deficits in at least two domains were classified as frail. A domain had deficits if at least one individual test score was abnormal

#### Blood sampling and metabolomics measurement

Non-fasting serum blood samples were collected at the time of inclusion, before treatment initiation, and stored at − 80 °C. The metabolomic biomarkers were measured in 100 µl serum samples by an external laboratory (Nightingale Health Ltd., Helsinki, Finland) using a high-throughput proton nuclear magnetic resonance (^1^H-NMR) metabolomics platform that is commercially available. The measurements were performed in March 2021 using the 2020 version of the quantification pipeline, an update to the 2014 version that was used to construct the metabolomics-based scores. This does not affect the MetaboHealth score and its behavior in associations, and the MetaboAge score was retrained [21]. The 1H-NMR metabolomics platform provides quantification of lipoprotein subclass profiling with lipid concentrations within 14 lipoprotein subclasses. The 14 subclass sizes were defined as follows: extremely large VLDL with particle diameters from 75 nm upwards and a possible contribution of chylomicrons, five VLDL subclasses (average particle diameters of 64.0 nm, 53.6 nm, 44.5 nm, 36.8 nm, and 31.3 nm), IDL (28.6 nm), three LDL subclasses (25.5 nm, 23.0 nm, and 18.7 nm), and four HDL subclasses (14.3 nm, 12.1 nm, 10.9 nm, and 8.7 nm). Within the lipoprotein subclasses, the following components were quantified: total cholesterol, total lipids, phospholipids, free cholesterol, cholesteryl esters, and triglycerides. The mean size for VLDL, LDL, and HDL particles was calculated by weighting the corresponding subclass diameters with their particle concentrations. Furthermore, 58 metabolic measures were determined that belong to classes of apolipoproteins, cholesterol, fatty acids, glycerides, phospholipids, amino acids, fluid balance, glycolysis-related metabolites, inflammation, and ketone bodies. A detailed description of the method used for the measurements is provided elsewhere [22]. The obtained set contained 250 biomarkers.

##### MetaboHealth

The MetaboHealth score was built by Deelen et al. as a linear model containing 14 metabolomic biomarkers [12]. The biomarkers were natural logarithmic transformed and then *z*-scaled to reduce the skewness of the distributions. In case of values under the detection limit, half of the minimum detectable value of the corresponding biomarker was added before transformation. The MetaboHealth score is the weighted sum of these values. MetaboHealth could not be calculated if one or more biomarkers were missing, which was the case in one patient due to a missing phenylalanine value. Details can be found in supplementary file, section A.

##### MetaboAge

The metabolomics-based age predictor MetaboAge was constructed by van den Akker et al. to predict a persons’ age and thereby obtain a biological age indication. The difference between chronological and predicted metabolomic age (MetaboAge) was defined as ΔMetaboAge and may reflect the discrepancy between biological and chronological age. A positive ΔMetaboAge indicates a relatively older blood metabolome compared to the chronological age and may indicate a higher biological age and mortality risk, validated in the study by van den Akker et al. We used the ElasticNET version of the new MetaboAge for this study; details on the development of MetaboAge are described elsewhere [13, 21, 23]. The model contains 63 biomarkers, listed in supplementary file, section A. Missing values (0.02% of all measurements) were imputed as zeros. The metabolomics features were *z*-scaled using the mean and standard deviations of the features in BBMRI-NL. ∆age was defined as MetaboAge minus the actual chronological age. ∆MetaboAge was calculated as the residuals signal of ∆age adjusted by chronological age. See supplementary file, section A for details on calculating ΔMetaboAge in our study.

##### Serum albumin

Albumin is one of the metabolomic biomarkers measured by the Nightingale platform, and is part of both metabolomics-based scores. To favor the clinical usefulness of biomarker measurements for daily practice, we also performed analyses with serum albumin. We chose to perform these analyses with albumin measured in the hospital laboratory of the LUMC instead of albumin measured in the metabolomics platform to copy daily clinical practice. Serum albumin was measured in batch and analyzed using a colorimetric assay (Roche Cobas 8000 Modular (C502)). The lower and upper limits of detection were 10 g/L and 336 g/L.

### Outcome

Outcome was all-cause 1-year mortality. Patients were followed 1 year after treatment initiation or until death. Data on mortality were obtained from digital patient files or municipal registries.

### Statistical methods

Baseline characteristics were expressed as numbers and percentages for categorical variables and mean (standard deviation (SD); or standard error (SE)) or median (interquartile range (IQR)) for continuous variables, depending on the distribution. Differences in metabolomics-based scores or individual metabolite levels between groups based on deficits in geriatric assessment or mortality status were compared by independent samples *T*-test or Mann–Whitney *U* test, as appropriate. Univariable Cox regression analyses were performed to study the association between MetaboHealth or ΔMetaboAge scores as continuous variables and all-cause 1-year mortality. Hazard ratios (HR) with 95% confidence intervals (CI) were calculated. The individual prediction accuracy of age, sex, primary tumor site, distant metastasis, Charlson Comorbidity Index, malnutrition, frailty, MetaboHealth, ΔMetaboAge, and serum albumin were tested for all-cause 1-year mortality using a receiver operating characteristic (ROC) curve with the area under the curve (AUC) assessed with Cox regression analyses. These predictors were chosen based on known predictors from clinical experience and statistical significance in univariable Cox regression analyses. MetaboHealth and ΔMetaboAge were combined with these predictors in multivariable Cox regression analyses and their AUCs were calculated and compared to assess whether the addition of the metabolomics-based scores improved the prediction accuracy. Comparison of the different AUCs was performed using the DeLong method [24] by MedCalc® Statistical Software version 20.115 (MedCalc Software Ltd., Ostend, Belgium; https://www.medcalc.org; 2022). A *p*-value of < 0.05 was considered statistically significant. Missing data are addressed in the table legends. Analyses were performed using SPSS software version 25.

## Results

A total of 488 patients with a solid malignant tumor were included in the TENT study between 2016 and 2020. Of 192 (39.3%) patients, a blood sample was available and included in this substudy; of 296 (60.7%) patients, no blood sample was available. Baseline characteristics of patients without a blood sample available are shown in Supplementary Table 1. There were slight differences in primary tumor site (most frequent: upper gastrointestinal tract cancers *n* = 67 (22.6%), gynecologic cancers *n* = 62 (20.9%), colorectal cancers *n* = 54 (18.2%)), and patients were less often classified as frail (62.1%) compared to the patients of which a blood sample was available.

### Baseline characteristics

Table 2 shows the baseline characteristics of the 192 patients that participated in the present study. Median age was 77 years (IQR 72.3–81.0) and 104 patients (54.2%) were male. Most common primary tumor sites were upper gastrointestinal tract cancers (*n* = 52, 27.1%), gynecologic cancers (*n* = 34, 17.7%), and colorectal cancers (*n* = 25, 13.0%). A total of 33 patients (17.2%) had metastatic disease. Most patients were treated with curative intent (*n* = 137, 71.4%). According to the geriatric assessment, 129 patients (68.3%) were frail. Among all patients, 98 (51.3%) were malnourished, 65 (34.8%) were dependent in their instrumental activities of daily living (IADL), and 29 (15.5%) had cognitive impairment. Data on biomarkers needed to calculate MetaboHealth was complete in 191 patients. ΔMetaboAge was calculated in all patients. The median MetaboHealth score was − 0.08 (IQR − 0.50–0.39) and the median ΔMetaboAge score was − 0.62 (IQR − 6.85–6.84).

**Table 2:** Baseline characteristics

Characteristics	Total population *n* = 192
Age, years, median (IQR)	77.0 (72.3–81.0)
Male gender, *n* (%)	104 (54.2)
BMI (kg/m^2^), mean (SD)	26.5 (4.9)
Smoking, history or current, *n* (%)	113 (58.9)
WHO PS, *n* (%)	
0–1	79 (41.1)
2	20 (10.4)
3–4	6 (3.1)
Unknown	87 (45.3)
Primary tumor site, *n* (%)	
Head and neck	19 (9.9)
Upper GI	52 (27.1)
Colorectal	25 (13.0)
Hepatobiliary or pancreas	9 (4.7)
Lung	10 (5.2)
Breast	16 (8.3)
Gynaecologic	34 (17.7)
Urologic	18 (9.4)
Other^a^	9 (4.7)
Distant metastasis, *n* (%)	
No	157 (81.8)
Yes	33 (17.2)
Unknown	2 (1.0)
Treatment intent, *n* (%)	
Curative	137 (71.4)
Palliative	55 (28.6)
Treatment plan, *n* (%)	
Surgery with/without (neo)adjuvant therapy	107 (55.7)
(Chemo)radiotherapy	35 (18.2)
No anti-tumor treatment	19 (9.9)
Chemotherapy	8 (4.2)
Other^b^	23 (12.0)
MetaboHealth, median (IQR)	− 0.08 (− 0.50–0.39)
ΔMetaboAge, median (IQR)	− 0.62 (− 6.85–6.84)
Geriatric assessment	
Frail, *n* (%)	129 (68.3)
Charlson Comorbidity index, median (IQR)	1.0 (0.0–3.0)
Number of medications, mean (SD)	5.8 (3.8)
Malnutrition, *n* (%)	98 (51.3)
Cognitive impairment according to 6CIT, *n* (%)	29 (15.5)
Dementia diagnosis, *n* (%)	3 (1.6)
Previous delirium, *n* (%)	27 (14.2)
ADL dependency, *n* (%)	9 (4.8)
IADL dependency, *n* (%)	65 (34.8)
Fall < 6 months, *n* (%)	46 (24.2)
Living situation, *n* (%)	
At home, alone	68 (35.4)
At home, with others	119 (62.0)
Institutionalized	4 (2.1)
Sheltered housing	1 (0.5)

*Abbreviation: 6CIT* Six-item Cognitive Impairment Test, *ADL* activities of daily living, *BMI* body mass index, *GI* gastrointestinal, *IADL* instrumental activities of daily living, *IQR* interquartile range, *n* number, *SD* standard deviation, *WHO PS* WHO performance score

^a^Other tumor sites: adrenal *n* = 1, bone *n* = 1, skin melanoma *n* = 2, soft tissue *n* = 3, thyroid *n* = 1, unknown primary tumor *n* = 1

^b^Other treatment: chemotherapy and immune therapy *n* = 2; chemotherapy and radiotherapy *n* = 4; chemotherapy and targeted therapy *n* = 2; chemotherapy, hormonal therapy, and targeted therapy *n* = 1; chemotherapy, radiotherapy, and immune therapy *n* = 1; hormonal therapy *n* = 5; immune therapy *n* = 2; radiofrequent ablation *n* = 1; radiotherapy and hormonal therapy *n* = 2; wait and see *n* = 3

Missing data: MetaboHealth *n* = 1, malnutrition *n* = 1, cognitive impairment *n* = 5, previous delirium *n* = 2, ADL dependency *n* = 3, IADL dependency *n* = 5, fall < 6 months *n* = 2, frail *n* = 3

### Metabolomics-based scores and geriatric assessment

Table 3 shows the median MetaboHealth and ΔMetaboAge scores for patients without deficits in geriatric assessment domains, compared to patients with deficits. Frail patients had a significantly higher MetaboHealth score (median 0.02 (IQR − 0.34–0.46)), compared to fit patients (median − 0.42 (IQR − 0.63–0.0), *p* < 0.01)). This effect is mainly explained by higher MetaboHealth scores in patients with deficits in the somatic and functional domains compared to patients with no deficits in these domains. ΔMetaboAge scores were not significantly different between patients without deficits, compared to patients with deficits. Results of the metabolomics-based scores and all individual items included in the geriatric assessment are described in Supplementary Table 2.

**Table 3:** Metabolomics-based scores and geriatric assessment

	Geriatric assessment result		*p* value^a^
	Fit (*n* = 60)	Frail (*n* = 129)	
MetaboHealth, median (IQR)	− 0.42 (− 0.63 to 0.20)	0.02 (− 0.34 to 0.46)	< 0.01
ΔMetaboAge, median (IQR)	− 1.94 (− 8.41 to 4.27)	0.15 (− 6.29 to 7.10)	0.17
	Somatic domain		
	No deficit (*n* = 18)	Deficit (*n* = 173)	
MetaboHealth, median (IQR)	− 0.61 (− 0.73 to − 0.21)	− 0.03 (− 0.45 to 0.46)	< 0.01
ΔMetaboAge, median (IQR)	− 3.97 (− 7.88 to 1.31)	0.15 (− 6.56 to 7.03)	0.18
	Psychological domain		
	No deficit (*n* = 139)	Deficit (*n* = 50)	
MetaboHealth, median (IQR)	− 0.08 (− 0.50 to 0.41)	− 0.07 (− 0.46 to 0.42)	0.82
ΔMetaboAge, median (IQR)	− 0.89 (− 6.87 to 6.12)	0.87 (− 7.36 to 8.08)	0.39
	Functional domain		
	No deficit (*n* = 102)	Deficit (*n* = 87)	
MetaboHealth, median (IQR)	− 0.33 (− 0.61 to 0.29)	0.06 (− 0.31 to 0.49)	< 0.01
ΔMetaboAge, median (IQR)	− 1.17 (− 7.96 to 5.12)	0.68 (− 6.22 to 7.97)	0.17
	Social domain		
	No deficit (*n* = 124)	Deficit (*n* = 68)	
MetaboHealth, median (IQR)	− 0.08 (− 0.50 to 0.47)	− 0.07 (− 0.46 to 0.30)	0.74
ΔMetaboAge, median (IQR)	0.38 (− 5.72 to 7.15)	− 1.09 (− 8.11 to 6.54)	0.33

*Abbreviation: IQR* interquartile range

^a^*p* value for difference in MetaboHealth or ΔMetaboAge between fit and frail patients, or patients with and without deficits in domains of the geriatric assessment, calculated with Mann–Whitney *U* test

Missing data: MetaboHealth *n* = 1 for all analyses, frailty *n* = 3, somatic domain *n* = 1, psychological domain *n* = 3, functional domain *n* = 3

### Metabolomics-based scores and all-cause 1-year mortality

Within 1 year, 58 patients (30.2%) died. Median MetaboHealth score was − 0.18 (IQR − 0.50–0.22) in patients that survived, compared to 0.19 (IQR − 0.29–0.81) in patients that died at 1 year of follow-up (*p* < 0.01). Median ΔMetaboAge score was − 1.25 (IQR − 7.84–6.07) in patients that survived, compared to 1.40 (IQR − 3.52–8.51) in patients that died (*p* = 0.04). Differences in single metabolite biomarkers between patients that survived and who died can be found in Supplementary Table 3 and 4. Among these, metabolic albumin levels were significantly lower in patients that died (*p* < 0.01). With each SD increase of MetaboHealth score, patients had a 2.32 times increased risk of mortality within the first year (HR 2.32, 95% CI 1.59–3.39). With each year of overestimation of chronological age (ΔMetaboAge), there was a 4% increased risk in mortality after 1 year (HR 1.04, 95% CI 1.01–1.07). Table 4 shows AUC for observed versus predicted all-cause 1-year mortality for clinical predictors and the metabolomics-based scores. Among the clinical predictors, primary tumor site had the highest AUC of 0.69 (95% CI 0.62–0.77). MetaboHealth had an AUC of 0.66 (95% CI 0.56–0.75) and ΔMetaboAge of 0.60 (95% CI 0.51–0.68). However, using the clinical predictors age, primary tumor site, presence of distant metastasis, CCI, and malnutrition together, the prediction of mortality was better (AUC 0.76; 95% CI 0.68–0.83). When combined with MetaboHealth, the performance marginally improved to an AUC of 0.80 (95% CI 0.74–0.87), but this increase was not statistically significant (*p* = 0.09). When combined with ΔMetaboAge, the performance did not change (AUC 0.76 (95% CI 0.69–0.83), *p* = 0.89).

**Table 4:** Area under the ROC curve for prediction of 1-year mortality

Predictors	AUC (95% CI)	*p* value^a^
Age	0.56 (0.47–0.65)	0.22
Sex	0.53 (0.44–0.62)	0.48
Primary tumor site	0.69 (0.62–0.77)	< 0.01
Distant metastasis	0.56 (0.47–0.65)	0.21
Charlson comorbidity index (CCI)	0.56 (0.47–0.65)	0.17
Malnutrition	0.60 (0.52–0.69)	0.03
Frailty	0.54 (0.45–0.63)	0.40
MetaboHealth	0.66 (0.56–0.75)	< 0.01
ΔMetaboAge	0.60 (0.51–0.68)	0.04
Serum albumin	0.65 (0.56–0.73)	< 0.01
Predictors combined		
Age + primary tumor site + distant metastasis + CCI + malnutrition	0.76 (0.68–0.83)	< 0.01
Age + primary tumor site + distant metastasis + CCI + malnutrition + MetaboHealth	0.80 (0.74–0.87)	< 0.01
Age + primary tumor site + distant metastasis + CCI + malnutrition + ΔMetaboAge	0.76 (0.69–0.83)	< 0.01
Age + primary tumor site + distant metastasis + CCI + malnutrition + serum albumin	0.79 (0.72–0.86)	< 0.01

*Abbreviation: AUC* area under the curve, *CCI* Charlson comorbidity index, *ROC* receiver operating characteristic curve

^a^*p* value for AUC comparison to reference line ROC curve

### Performance check single biomarker: serum albumin

The Pearson correlation between albumin measured by the Nightingale platform and serum albumin measured in the LUMC laboratory was 0.86. Serum albumin alone had an AUC of 0.65 (95% CI 0.56–0.73) for mortality prediction, which was similar to the performance of MetaboHealth (*p* = 0.98) and ΔMetaboAge (*p* = 0.15). Combining albumin with the clinical predictors resulted in an AUC of 0.79 (95% CI 0.72–0.86), also similar to the clinical predictors together with MetaboHealth (*p* = 0.75) and marginally better than the performance of clinical predictors combined with ΔMetaboAge, but not statistically significant (*p* = 0.07).

## Discussion

The main findings of the current study are threefold. First, a higher MetaboHealth score was associated with frailty, explained by deficits in the somatic and functional domain, indicating that the MetaboHealth score is indicative of general health in older patients with cancer. MetaboAge was not significantly associated with frailty. Second, higher MetaboHealth and MetaboAge scores were associated with an increased all-cause 1-year mortality risk in our cohort of older patients with solid tumors. Third, addition of MetaboHealth to established clinical predictors marginally improved mortality prediction in this cohort and addition of MetaboAge resulted in no improvement.

To the best of our knowledge, no previous studies investigated the association of MetaboHealth and MetaboAge with geriatric deficits and mortality in older patients with cancer. Kuiper et al. studied the association of MetaboHealth and MetaboAge with five different frailty scores in population-based cohorts in the Netherlands [14]. Of these five frailty scores, the Multidimensional Prognostic Index (MPI) most closely resembles our frailty definition as it is also based on the domains of the Comprehensive Geriatric Assessment and contains overlapping tests within the geriatric domains. Kuiper et al. found that both metabolomics-based scores were associated with frailty using the MPI. The association was stronger for MetaboHealth than for MetaboAge. In our study, only a higher MetaboHealth was associated with frailty. Previous studies in older patients with cancer showed that other metabolomics-based scores predicted cancer recurrence in colorectal cancer [25] and breast cancer [26]. Van der Sijp et al. studied the performance of MetaboHealth in older patients with a femoral fracture presenting to the emergency department and found a 2.74 times higher mortality risk for every unit increase in MetaboHealth score. In line with our results, accuracy of mortality prediction showed an AUC of 0.68 (95% CI 0.56–0.81) [15]. The development and validation study by Deelen et al. also showed a 2.73 times higher mortality risk for every SD increase (HR, 2.73; 95% CI 2.60–2.86). The AUC for 5-year mortality was 0.84 and for 10-year mortality 0.83 in their mortality prediction analyses in one of the cohorts (the Estonian Biobank cohort) [12]. The study by Deelen et al. was very different in study population, since the cohorts included were population-based cohorts with a lower mean age at inclusion compared to the median age of 77 years in our study. Although the only provided baseline characteristics of the cohorts were age and sex, people in these population-based cohorts were presumably much healthier compared to the older patients in our cohort, presenting with solid tumors with a high prevalence of comorbidity, malnutrition, and frailty. The mortality risk is higher in older patients with cancer compared to the adults in these population cohorts due to cancer-related risk factors that might not be captured by the MetaboHealth score. Also, MetaboHealth was developed for 5- and 10-year mortality in cohorts with a much lower mortality rate (12.5%, mean follow-up time ranging from 2.76 to 16.70 years) [12]. Interestingly, despite the differences in study population and mortality rates, MetaboHealth showed a similar association and modest prediction. MetaboAge was developed in a dataset derived from 26 population-based and hospital-based cohorts that probably also contained people that were healthier compared to our cohort. In the majority of the cohorts, the age at blood sample collection was ≤ 70 years [13]. Still, similar to our results, a high MetaboAge score was associated with mortality in the development and validation study by Van den Akker et al.

To test our hypothesis whether metabolomics-based scores were also associated with correlates of early mortality, like geriatric deficits, we compared metabolomics-based scores between patients with and without deficits in geriatric assessment. We demonstrated that deficits in the somatic and functional domain but not the psychological and social domains were associated with higher MetaboHealth scores. These findings can help to understand the association of the score with mortality, as the scores might be a proxy for general health or biological age, as also discussed by Deelen and van den Akker. However, while MetaboAge is developed as biological age predictor, the geriatric deficits or general health parameters were not significantly associated with MetaboAge. A possible explanation might be that biomarker scores trained on mortality information seem to outperform biomarker scores trained on age in the association with frailty [14].

MetaboHealth and MetaboAge had moderate and poor prediction accuracy for all-cause 1-year mortality in older patients with solid tumors in the current study. The prediction accuracy was similar to single established clinical predictors, such as primary tumor site. A score based on cancer associated metabolites [8] may be more informative in that sense than the generic scores tested here, which were shown to be more indicative for cardiometabolic health than cancer. Remarkably, some of the established clinical predictors poorly predicted all-cause 1-year mortality in this cohort, such as the presence of distant metastasis and frailty. This might be explained by the enormous heterogeneity of the cohort in terms of tumor type and treatment, and the high prevalence of frailty due to the selection of patients.

For biomarkers to be of additional value in clinical practice, they should improve identification of older patients with high risk of early mortality and complications. This study suggests that MetaboHealth has the potential to provide prognostic information, with similar performance as clinical predictors and a non-significant improve in prediction accuracy of all-cause 1-year mortality when combined with clinical predictors. As also described in our previous work (*reference to van Holstein Y, Trompet S, van Munster B.C. *et al*. Association of Glasgow Prognostic Score with frailty, mortality and adverse health outcomes in older patients with cancer; submitted for publication at the Journal of Geriatric Oncology*), serum albumin alone reached similar prediction accuracy in the current cohort, while it is already available in clinical practice, easier to measure, and therefore a more practical measurement right now. We chose to analyze albumin separately since it was the only biomarker routinely measured in clinical practice that is included in both metabolomics-based scores that was significantly different between patients that survived and died within 1 year in this cohort. For MetaboHealth to be incorporated in clinical practice, it should be readily available and easy to measure. Currently, these criteria are not met, since the calculation score imposes the use of blood biomarker values scaled in a research cohort, consequently conferring a value per individual in relation to a certain dataset, and because readily available measured albumin gives similar results. However, the development of technical applications to calculate an individual MetaboHealth score [23] and the use of NMR facilities in (academic) hospitals contribute to potential use of metabolomics-based scores in clinical practice in the future. Improving the metabolomics-based biomarker scores by metabolites measured at absolute values is currently work in progress in the Vitality Oriented Innovations for the Lifecourse of the Ageing Society (VOILA) project. The costs of metabolomics measurements do not exceed routine measurements in hospital laboratories. Therefore, it is worthwhile to further investigate the performance of MetaboHealth, possibly improved by the addition of cancer-related metabolites, as a prognostic tool in larger and more homogeneous patient groups with similar tumor types and treatment regimens with data available on geriatric assessment and routinely available blood biomarkers. Also, alternative outcomes that are less subject to treatment choice and intent are of interest, such as treatment complications or toxicity.

The present study has some limitations. First, only 39% of patients included in the TENT study had blood samples available for metabolomics measurements. However, patients without a blood sample available were only slightly different in primary tumor site and had a lower frailty prevalence. Second, the sample size was relatively small and patients were heterogeneous in tumor type and treatment, which both influence prognosis. The small sample size hampered stratification for tumor type. Third, almost 70% of patients were frail. This could have diminished the effect of frailty as mortality predictor. Strengths of the current study include the prospective study design, the assessment of all geriatric assessment domains, and the inclusion of an older population with cancer seen in daily routine care. Finally, this is the first study that investigated the performance of these metabolomics-based scores in older patients with cancer.

## Conclusion

Higher MetaboHealth and MetaboAge scores were associated with 1-year mortality. The addition of MetaboHealth to established clinical predictors only marginally improved mortality prediction in this cohort with various types of tumors. MetaboHealth may potentially improve identification of older patients vulnerable for adverse events, but numbers were too small for definitive conclusions.

## Supplementary Information

Below is the link to the electronic supplementary material.

Supplementary file1 (DOCX 133 KB)

## Data Availability

The data that support the findings of this study are available on reasonable request from the corresponding author, YvH.
