# Supplementary material for: The performance of metabolomics-based prediction scores for mortality in older patients with solid tumors
Source: GeroScience. 2024 Jul 4;46(6):5615–27. doi: 10.1007/s11357-024-01261-6 (PMC11493906; doi:10.1007/s11357-024-01261-6)
Supplement: Supplementary file 1 — Supplementary file1 (DOCX 133 KB) [file 11357_2024_1261_MOESM1_ESM.docx]

**Supplementary information**

**The performance of metabolomics-based prediction scores for mortality in older patients with solid tumors**

Yara van Holstein ^1^; Simon P. Mooijaart ^1,2^; Mathijs van Oevelen ^3^; Floor J. van Deudekom ^4^; Dina Vojinovic ^5,6^; Daniele Bizzarri ^5,7^; Erik B. van den Akker ^5,7^ Raymond Noordam ^1^; Joris Deelen ^8,9^; Diana van Heemst ^1^; Nienke A. de Glas ^10^; Cynthia Holterhues ^11^; Geert Labots ^11^; Frederiek van den Bos ^1^; Marian Beekman ^5^; P. Eline Slagboom ^5^; Barbara C. van Munster ^12^; Johanneke E.A. Portielje ^10^; Stella Trompet ^1^

^1^ Department of Internal Medicine, section of Gerontology and Geriatrics, Leiden University Medical Center, Leiden, The Netherlands

^2^ LUMC Center for Medicine for Older People, Leiden University Medical Center, Leiden, The Netherlands

^3^ Department of Internal Medicine, section of Nephrology, Leiden University Medical Center, Leiden, The Netherlands

^4^ Department of Geriatric Medicine, OLVG Hospitals Amsterdam, Amsterdam, The Netherlands

^5^ Department of Biomedical Data Sciences, section of Molecular Epidemiology, Leiden University Medical Center, Leiden, The Netherlands

^6^ Department of Epidemiology, Erasmus Medical Center, University Medical Centre, Rotterdam, The Netherlands

^7^ Delft Bioinformatics Lab, Delft University of Technology, Delft, The Netherlands

^8^ Max Planck Institute for Biology of Ageing, Cologne, Germany

^9^ Cologne Excellence Cluster on Cellular Stress Responses in Ageing-Associated Diseases (CECAD), University of Cologne, Cologne, Germany

^10^ Department of Medical Oncology, Leiden University Medical Center, Leiden, The Netherlands

^11^ Department of Internal Medicine, Haga Hospital, The Hague, The Netherlands

^12^ Department of Internal Medicine, University Medical Center Groningen, Groningen, The Netherlands

**Corresponding author:** Yara van Holstein, Department of Internal Medicine, section of Gerontology and Geriatrics, Leiden University Medical Center, PO box 9600, 2300 RC Leiden, The Netherlands. Telephone: +3171-5266640. E-mail: [Y.van_Holstein@lumc.nl](mailto:Y.van_Holstein@lumc.nl)

**Content**

Supplementary file, section A: Calculation of metabolomics-based scores.

Supplementary table 1. Baseline characteristics patients with and without blood sample available.

Supplementary table 2. Metabolomics-based scores and tests geriatric assessment.

Supplementary table 3. Single metabolic biomarkers and MetaboHealth by mortality status.

Supplementary table 4. Single metabolic biomarkers and ΔMetaboAge by mortality status.

Supplementary figure 1. Correlation between albumin measured by the Nightingale platform and LUMC laboratory.

**Calculation of metabolomics-based scores**

*MetaboHealth*

MetaboHealth = (((Z(ln[XXL_VLDL_L]))*ln(0.80)) + ((Z(ln[S_HDL_L]))*ln(0.87)) + ((Z(ln[VLDL-D]))*ln(0.85)) + ((Z(ln[PUFA/FA]))*ln(0.78)) + ((Z(ln[Glucose]))*ln(1.16)) +

((Z(ln[Lactate]))*ln(1.06)) + ((Z(ln[Histidine]))*ln(0.93)) + ((Z(ln[Isoleucine]))*ln(1.23)) + ((Z(ln[Leucine]))*ln(0.82)) + ((Z(ln[Valine]))*ln(0.87)) + ((Z(ln[Phenylalanine]))*ln(1.13)) + ((Z(ln[Acetoacetate]))*ln(1.08)) + ((Z(ln[Albumin]))*ln(0.89)) + ((Z(ln[Glycoprotein_acetyls]))*ln(1.32))).

Z states for z-scaling and ln states for natural logarithm.

*MetaboAge*

Exclude samples if more than one metabolic feature is missing, more than one metabolic feature is reported as zero or if a metabolic feature is 5 SD away from the mean in BBMRI-NL. Remaining samples are imputed as zeros.

Predicted age (MetaboAge) =

59.22650 + Z(Acetoacetate BBMRI) * 1.517445 + Z(Acetate BBMRI) * 0.5313667 + Z(Alanine BBMRI) * 0.6596228 + Z(Albumin BBMRI) * -3.482258 + Z(ApoA1 BBMRI) * 42.43577 + Z(ApoB BBMRI) * -21.18887 + Z(Creatinine BBMRI) * 2.067322 + Z(DHA BBMRI) * 1.051034 + Z(Omega-3 FA BBMRI) * 0.9965705 + Z(Omega-3/FA BBMRI) * 7.366434 + Z(Omega-6 FA BBMRI) * 16.7692 + Z(Omega-6/FA BBMRI) * 12.32999 + Z(Glucose BBMRI) * 2.042961 + Z(Glutamine BBMRI) * 4.344563 + Z(Glycoprotein acetyls BBMRI) * -0.5617847 + Z(HDL_C BBMRI) * -7.293456 + Z(HDL_D BBMRI) * 0.9977565 + Z(Histidine BBMRI) * -1.845607 + Z(IDL_C BBMRI) * 15.58729 + Z(IDL_L BBMRI) * -12.04912 + Z(Isoleucine BBMRI) * 1.691601 + Z(L_HDL_C BBMRI) * -46.32489 + Z(L_HDL_L BBMRI) * 69.49613 + Z(L_LDL_L BBMRI) * -37.324787 +

Z(L_VLDL_L BBMRI) * 74.32913 + Z(LA BBMRI) * 5.969971 + Z(Lactate BBMRI) * 3.144457 +

Z(LDL_C BBMRI) * 59.38953 + Z(LDL_D BBMRI) * -1.762851 + Z(Leucine BBMRI) * -5.252610 +

Z(M_HDL_C BBMRI) * 31.15148 + Z(M_HDL_L BBMRI) * -80.62610 + Z(M_LDL_L BBMRI) * -32.45705 + Z(M_VLDL_L BBMRI) * -29.420457 + Z(MUFA BBMRI) * 1.064991 + Z(MUFA_FA BBMRI) * 1.363744 + Z(Phosphatidylcholines BBMRI) * -4.402335 + Z(Phenylalanine BBMRI) * 2.739373 + Z(PUFA BBMRI) * -23.64967 + Z(PUFA _FA BBMRI) * -10.21774 + Z(Pyruvate BBMRI) * -0.1951973 + Z(S_HDL_C BBMRI) * -0.3228061 + Z(S_HDL_L BBMRI) * 18.124134 + Z(S_LDL_L BBMRI) * 17.84038 + Z(S_VLDL_L BBMRI) * 28.82002 + Z(Total cholesterol BBMRI) * 6.457966 + Z(Total triglycerides BBMRI) * -104.7649 +

Z(SFA BBMRI) * 12.20618 + Z(SFA_FA BBMRI) * 2.396089 + Z(Sphingomyelins BBMRI) * -11.04818 +

Z(Total cholines BBMRI) * -22.2124 + Z(Total fatty acids BBMRI) * 21.87194 + Z(Phosphoglycerides BBMRI) * -1.266618 x 10^-03^ + Z(Tyrosine BBMRI) * 3.056520 + Z(Unsaturation BBMRI) * 4.342435 +

Z(Valine BBMRI) * 2.388533 + Z(VLDL_C BBMRI) * -34.81194 + Z(VLDL_D BBMRI) * 3.22391 +

Z(XL_HDL_C BBMRI) * 3.471796 + Z(XL_HDL_L BBMRI) * -12.015792 + Z(XL_VLDL_L BBMRI) * 22.89381 + Z(XS_VLDL_L BBMRI) * 35.62201 + Z(XXL_VLDL_L BBMRI) * 7.436942

Δage = MetaboAge – chronological age. ΔMetaboAge was calculated as the residuals signal of ∆age adjusted by chronological age.

**Supplementary table 1. Baseline characteristics patients with and without blood sample available.**

| **Characteristics** | **Patients with blood sample (n=192)** | **Patients without blood sample (n=296)** |
| --- | --- | --- |
| Age, years, median (IQR) | 77.0 (72.3-81.0) | 77.0 (73.0-81.0) |
| Male gender, n (%) | 104 (54.2) | 152 (51.4) |
| BMI (kg/m^2^), mean (SD) | 26.5 (4.9) | 25.5 (4.4) |
| Smoking, history or current, n (%) | 113 (58.9) | 169 (57.5) |
| WHO PS, n (%) |  |  |
| 0-1 | 79 (41.1) | 117 (39.6) |
| 2 | 20 (10.4) | 34 (11.5) |
| 3-4 | 6 (3.1) | 9 (3.0) |
| Unknown | 87 (45.3) | 136 (45.9) |
| Tumor site, n (%) |  |  |
| Head and neck | 19 (9.9) | 48 (16.2) |
| Upper GI | 52 (27.1) | 67 (22.6) |
| Lower GI | 25 (13.0) | 54 (18.2) |
| Hepatobiliary or pancreas | 9 (4.7) | 13 (4.4) |
| Lung | 10 (5.2) | 11 (3.7) |
| Breast | 16 (8.3) | 10 (3.4) |
| Gynecologic | 34 (17.7) | 62 (20.9) |
| Urologic | 18 (9.4) | 24 (8.1) |
| Other^a^ | 9 (4.7) | 7 (2.4) |
| Distant metastasis, n (%) |  |  |
| No | 157 (81.8) | 230 (77.7) |
| Yes | 33 (17.2) | 49 (16.6) |
| Unknown | 2 (1.0) | 17 (5.7) |
| **Geriatric assessment** |  |  |
| Frail, n (%) | 129 (68.3) | 175 (62.1) |
| Charlson comorbidity index, median (IQR) | 1.0 (0.0-3.0) | 1.0 (0.0-2.0) |
| Number of medication, mean (SD) | 5.8 (3.8) | 5.0 (3.6) |
| Malnutrition, n (%) | 98 (51.3) | 138 (48.6) |
| Cognitive impairment according to 6CIT, n (%) | 29 (15.5) | 20 (7.0) |
| Dementia diagnosis, n (%) | 3 (1.6) | 2 (0.7) |
| Previous delirium, n (%) | 27 (14.2) | 26 (8.9) |
| ADL dependency, n (%) | 9 (4.8) | 11 (3.8) |
| IADL dependency, n (%) | 65 (34.8) | 72 (25.6) |
| Fall <6 months, n (%) | 46 (24.2) | 66 (22.8) |
| Living situation, n (%) |  |  |
| At home, alone | 68 (35.4) | 108 (36.5) |
| At home, with others | 119 (62.0) | 181 (61.1) |
| Institutionalized | 4 (2.1) | 4 (1.4) |
| Sheltered housing | 1 (0.5) | 3 (1.0) |

*Abbreviations: 6CIT, Six-item Cognitive Impairment Test; ADL, Activities of Daily Living; BMI, Body mass index; GI, Gastrointestinal; IADL, Instrumental Activities of Daily Living; IQR, Interquartile range; n, number; SD, standard deviation; WHO PS, WHO performance score.*

*^a^Other tumor sites: Patients with blood sample: Adrenal n=1, bone n=1, skin melanoma n=2, soft tissue n=3, thyroid n=1, unknown primary tumor n=1. Patients without blood sample: Bone n=1, brain n=1, eye n=1, skin melanoma n=2, soft tissue n=2.*

*Missing data: Patients with blood sample: frail n=3, malnutrition n=1, cognitive impairment n=5, previous delirium n=2, ADL dependency n=3, IADL dependency n=5, fall <6 months n=2.*

*Patients without blood sample: Smoking n=2, frail n=14, malnutrition n=12, cognitive impairment n=12, previous delirium n=3, dementia diagnosis n=3, ADL n=3, IADL n=15, fall <6 months n=6.*

**Supplementary table 2. Metabolomics-based scores and tests geriatric assessment.**

|  | **Geriatric assessment result** | | **P value ^a^** |
| --- | --- | --- | --- |
|  | **Somatic domain** | |  |
|  | **CCI 0** | **CCI ≥1** |  |
| MetaboHealth, median (IQR) | -0.33 (-0.62-0.18) | -0.02 (-0.43-0.45) | 0.03 |
| ΔMetaboAge, median (IQR) | -1.12 (-7.48-8.11) | -0.18 (-6.36-6.76) | 0.54 |
|  | **No polypharmacy** | **Polypharmacy** |  |
| MetaboHealth, median (IQR) | -0.33 (-0.61-0.39) | 0.00 (-0.38-0.41) | 0.02 |
| ΔMetaboAge, median (IQR) | -2.48 (-8.58-5.85) | 0.67 (-5.49-7.14) | 0.07 |
|  | **No malnutrition** | **Malnutrition** |  |
| MetaboHealth, median (IQR) | -0.32 (-0.61-0.15) | 0.14 (-0.35-0.63) | <0.01 |
| ΔMetaboAge, median (IQR) | -1.60 (-7.21-3.74) | 0.96 (-6.25-8.40) | 0.13 |
|  | **Psychological domain** | |  |
|  | **No cognitive impairment (6CIT)** | **Cognitive impairment (6CIT)** |  |
| MetaboHealth, median (IQR) | -0.08 (-0.50-0.40) | -0.04 (-0.28-0.42) | 0.42 |
| ΔMetaboAge, median (IQR) | -1.08 (-7.12-6.07) | 1.06 (-3.35-7.85) | 0.38 |
|  | **No dementia diagnosis** | **Dementia diagnosis** |  |
| MetaboHealth, median (IQR) | -0.08 (-0.49-0.39) | -0.03 (-0.58-NR) | 0.93 |
| ΔMetaboAge, median (IQR) | -0.65 (-6.83-6.80) | 0.15 (-8.12-NR) | 0.72 |
|  | **No previous delirium** | **Previous delirium** |  |
| MetaboHealth, median (IQR) | -0.05 (-0.47-0.41) | -0.18 (-0.52-0.31) | 0.36 |
| ΔMetaboAge, median (IQR) | -0.60 (-6.35-6.67) | 0.68 (-8.12-8.39) | 0.79 |
|  | **Functional domain** | |  |
|  | **ADL independent** | **ADL dependent** |  |
| MetaboHealth, median (IQR) | -0.08 (-0.50-0.44) | 0.02 (-0.18-0.30) | 0.64 |
| ΔMetaboAge, median (IQR) | -0.96 (-7.16-6.54) | 4.82 (-1.33-11.79) | 0.40 |
|  | **IADL independent** | **IADL dependent** |  |
| MetaboHealth, median (IQR) | -0.23 (-0.57-0.38) | 0.07 (-0.18-0.45) | <0.01 |
| ΔMetaboAge, median (IQR) | -1.25 (-8.29-5.12) | 0.68 (-5.05-8.79) | 0.06 |
|  | **No fall <6 months** | **Fall <6 months** |  |
| MetaboHealth, median (IQR) | -0.15 (-0.54-0.36) | 0.07 (-0.32-0.69) | 0.02 |
| ΔMetaboAge, median (IQR) | -0.62 (-6.47-6.10) | 0.96 (-7.36-8.20) | 0.68 |
|  | **Social domain** | |  |
|  | **Not living at home alone** | **Living at home, alone** |  |
| MetaboHealth, median (IQR) | -0.08 (-0.50-0.47) | -0.07 (-0.46-0.30) | 0.74 |
| ΔMetaboAge, median (IQR) | 0.38 (-5.72-7.15) | -1.09 (-8.11-6.54) | 0.33 |
|  | **Not living at home with others** | **Living at home, with others** |  |
| MetaboHealth, median (IQR) | -0.08 (-0.49-0.28) | -0.08 (-0.50-0.50) | 0.49 |
| ΔMetaboAge, median (IQR) | -1.09 (-8.18-6.40) | 0.62 (-5.46-7.15) | 0.29 |
|  | **Not institutionalized** | **Institutionalized** |  |
| MetaboHealth, median (IQR) | -0.08 (-0.49-0.41) | -0.26 (-0.84-0.28) | 0.42 |
| ΔMetaboAge, median (IQR) | -0.55 (-6.47-6.84) | -7.87 (-8.34-16.97) | 0.50 |
|  | **Not living in sheltered housing** | **Living in sheltered housing** |  |
| MetaboHealth, median (IQR) | -0.08 (-0.50-0.40) | -0.47 (N/A) | 0.52 |
| ΔMetaboAge, median (IQR) | -0.65 (-6.87-6.87) | 2.85 (N/A) | 0.64 |

*Abbreviations: 6CIT, Six-item Cognitive Impairment Test; ADL, Activities of Daily Living; CCI, Charlson Comorbidity Index; IADL, Instrumental Activities of Daily Living; IQR, Interquartile range.*

*^a^ P value for difference in MetaboHealth or ΔMetaboAge between patients without and with deficits in geriatric tests, calculated with Mann-Whitney U test.
Missing data: Metabohealth n=1 for all analyses, malnutrition n=1, 6CIT n=5, previous delirium n=2, ADL n=3, IADL n=5, fall <6 months n=2.*

**Supplementary table 3. Single metabolic biomarkers and MetaboHealth by mortality status.**

| **Blood metabolites** | **Alive n=134** | **Dead n=58** | **P value** |
| --- | --- | --- | --- |
| XXL-VLDL-L | 0.13 (0.04-0.32) | 0.10 (0.03-0.23) | 0.29 |
| S-HDL-L | 1.14 (0.02) | 1.04 (0.02) | <0.01 |
| VLDL-D (nm) | 38.68 (0.11) | 38.24 (0.19) | 0.04 |
| PUFA/FA (%) | 41.61 (39.04-43.76) | 41.13 (38.77-43.63) | 0.87 |
| Glucose | 5.66 (5.04-7.10) | 5.84 (5.08-7.10) | 0.66 |
| Lactate | 2.08 (1.79-2.57) | 2.15 (1.67-2.56) | 0.65 |
| Histidine | 0.08 (0.00) | 0.07 (0.00) | <0.01 |
| Isoleucine | 0.06 (0.05-0.08) | 0.06 (0.04-0.06) | 0.04 |
| Leucine | 0.12 (0.10-0.15) | 0.11 (0.09-0.13) | 0.12 |
| Valine | 0.24 (0.21-0.29) | 0.23 (0.20-0.27) | 0.08 |
| Phenylalanine | 0.08 (0.07-0.09) | 0.09 (0.07-0.10) | 0.07 |
| Acetoacetate | 0.01 (0.01-0.03) | 0.02 (0.01-0.03) | 0.79 |
| Albumin (g/l) | 38.63 (35.79-41.57) | 36.44 (31.90-39.66) | <0.01 |
| Glycoprotein acetyls | 1.00 (0.02) | 1.05 (0.03) | 0.11 |
| MetaboHealth | -0.18 (-0.50-0.22) | 0.19 (-0.29-0.81) | <0.01 |

*Abbreviations: nm, nanometer; PUFA/FA, Ratio of polyunsaturated fatty acids to total fatty acids; S-HDL-L, Total lipids in small HDL; VLDL-D, Mean diameter for VLDL particles; XXL-VLDL-L, Total lipids in chylomicrons and extremely large VLDL.*

*Concentrations are presented in mmol/l unless stated otherwise.
Mean (SE mean), median (IQR). Independent samples T-test or Mann-Whitney U test.*
*Missing data: Dead: Phenylalanine n=1, MetaboHealth n=1.*

**Supplementary table 4. Single metabolic biomarkers and ΔMetaboAge by mortality status.**

| **Blood metabolites** | **Alive n=134** | **Dead n=58** | **P value** |
| --- | --- | --- | --- |
| M-VLDL-L | 0.55 (0.39-0.73) | 0.51 (0.35-0.65) | 0.04 |
| S-VLDL-L | 0.44 (0.01) | 0.41 (0.02) | 0.17 |
| XS-VLDL-L | 0.33 (0.29-0.41) | 0.32 (0.28-0.39) | 0.57 |
| IDL-L | 1.15 (0.92-1.37) | 1.05 (0.87-1.21) | 0.05 |
| IDL-C | 0.77 (0.60-0.93) | 0.69 (0.54-0.79) | 0.03 |
| L-LDL-L | 1.58 (1.22-1.86) | 1.41 (1.08-1.61) | 0.01 |
| M-LDL-L | 0.63 (0.50-0.76) | 0.56 (0.43-0.67) | 0.02 |
| M-HDL-L | 0.96 (0.85-1.17) | 0.90 (0.77-1.05) | 0.03 |
| S-HDL-L | 1.14 (0.02) | 1.04 (0.02) | <0.01 |
| S-LDL-L | 0.28 (0.24-0.34) | 0.26 (0.21-0.31) | 0.04 |
| VLDL-D (nm) | 38.68 (0.11) | 38.24 (0.19) | 0.04 |
| LDL-D (nm) | 23.93 (23.88-23.99) | 23.96 (23.89-24.00) | 0.11 |
| HDL-D (nm) | 9.61 (9.50-9.76) | 9.63 (9.49-9.82) | 0.48 |
| Total cholesterol | 4.47 (3.59-5.12) | 4.02 (3.39-4.51) | 0.01 |
| VLDL cholesterol | 0.66 (0.47-0.81) | 0.59 (0.44-0.77) | 0.18 |
| LDL cholesterol | 1.74 (1.31-2.06) | 1.53 (1.14-1.75) | 0.01 |
| HDL cholesterol | 1.24 (1.04-1.49) | 1.20 (1.04-1.44) | 0.32 |
| Total triglycerides | 1.26 (0.93-1.87) | 1.15 (0.88-1.65) | 0.18 |
| Phosphoglycerides | 2.31 (2.02-2.65) | 2.09 (1.94-2.34) | 0.01 |
| Phosphatidylcholines | 2.15 (1.88-2.47) | 1.92 (1.76-2.14) | <0.01 |
| Sphingomyelins | 0.46 (0.41-0.52) | 0.43 (0.38-0.48) | 0.10 |
| Total cholines | 2.61 (0.04) | 2.43 (0.06) | 0.02 |
| ApoA1 (g/l) | 1.40 (1.25-1.60) | 1.33 (1.17-1.50) | 0.03 |
| ApoB (g/l) | 0.81 (0.67-0.98) | 0.76 (0.61-0.90) | 0.09 |
| Total fatty acids | 12.65 (10.61-14.52) | 11.44 (10.55-13.23) | 0.03 |
| Degree of unsaturation (degree) | 1.32 (1.28-1.36) | 1.32 (1.27-1.38) | 0.90 |
| DHA | 0.25 (0.22-0.30) | 0.24 (0.21-0.28) | 0.26 |
| Linoleic acid (LA) | 3.75 (3.13-4.25) | 3.42 (2.94-3.83) | 0.01 |
| Omega-3 fatty acids | 0.54 (0.43-0.70) | 0.53 (0.41-0.64) | 0.51 |
| Omega-6 fatty acids | 4.63 (4.06-5.14) | 4.21 (3.82-4.75) | 0.01 |
| PUFA | 5.17 (4.55-5.83) | 4.78 (4.22-5.32) | 0.02 |
| MUFA | 3.14 (2.43-3.84) | 2.88 (2.49-3.41) | 0.21 |
| Saturated fatty acids (SFA) | 4.20 (3.59-5.02) | 3.87 (3.50-4.33) | 0.03 |
| Omega-3/FA (%) | 4.43 (3.77-5.15) | 4.49 (3.78-5.18) | 0.51 |
| Omega-6/FA (%) | 36.90 (34.76-39.46) | 36.01 (34.37-38.82) | 0.37 |
| PUFA/FA (%) | 41.61 (39.04-43.76) | 41.13 (38.77-43.63) | 0.87 |
| MUFA/FA (%) | 24.87 (23.14-26.43) | 24.58 (23.23-27.27) | 0.87 |
| SFA/FA (%) | 34.04 (32.63-35.00) | 33.86 (32.34-35.31) | 0.64 |
| Glucose | 5.66 (5.04-7.10) | 5.84 (5.08-7.10) | 0.66 |
| Lactate | 2.08 (1.79-2.57) | 2.15 (1.67-2.56) | 0.65 |
| Alanine | 0.44 (0.39-0.50) | 0.42 (0.36-0.50) | 0.19 |
| Glutamine | 0.60 (0.55-0.68) | 0.57 (0.49-0.63) | 0.01 |
| Histidine | 0.08 (0.00) | 0.07 (0.00) | <0.01 |
| Isoleucine | 0.06 (0.05-0.08) | 0.06 (0.04-0.06) | 0.04 |
| Leucine | 0.12 (0.10-0.15) | 0.11 (0.09-0.13) | 0.12 |
| Valine | 0.24 (0.21-0.29) | 0.23 (0.20-0.27) | 0.08 |
| Phenylalanine | 0.08 (0.07-0.09) | 0.09 (0.07-0.10) | 0.07 |
| Tyrosine | 0.07 (0.06-0.08) | 0.06 (0.05-0.08) | 0.63 |
| Acetate | 0.02 (0.02-0.04) | 0.02 (0.02-0.03) | 0.53 |
| Acetoacetate | 0.01 (0.01-0.03) | 0.02 (0.01-0.03) | 0.79 |
| Creatinine (µmol/l) | 80.57 (70.96-94.99) | 76.88 (68.05-95.82) | 0.44 |
| Albumin (g/l) | 38.63 (35.79-41.57) | 36.44 (31.90-39.66) | <0.01 |
| Glycoprotein acetyls | 1.00 (0.02) | 1.05 (0.03) | 0.11 |
| L-HDL-C | 0.27 (0.17-0.37) | 0.27 (0.17-0.40) | 0.80 |
| L-HDL-L | 0.59 (0.42-0.77) | 0.58 (0.42-0.83) | 0.99 |
| L-VLDL-L | 0.30 (0.19-0.51) | 0.27 (0.16-0.41) | 0.08 |
| M-HDL-C | 0.46 (0.39-0.56) | 0.44 (0.37-0.53) | 0.08 |
| Pyruvate | 0.06 (0.04-0.08) | 0.06 (0.04-0.08) | 0.74 |
| S-HDL-C | 0.44 (0.40-0.49) | 0.41 (0.37-0.45) | <0.01 |
| XL-HDL-C | 0.08 (0.06-0.10) | 0.08 (0.06-0.11) | 0.28 |
| XL-HDL-L | 0.15 (0.11-0.20) | 0.16 (0.12-0.22) | 0.21 |
| XL-VLDL-L | 0.18 (0.10-0.31) | 0.15 (0.09-0.25) | 0.11 |
| XXL-VLDL-L | 0.13 (0.04-0.32) | 0.10 (0.03-0.23) | 0.29 |
| Predicted MetaboAge | 66.0 (59.8-73.0) | 68.5 (63.2-75.6) | 0.03 |
| ΔMetaboAge | -1.25 (-7.84-6.07) | 1.40 (-3.52-8.51) | 0.04 |

*Abbreviations: ApoA1, Apolipoprotein A-I; ApoB, Apolipoprotein B; DHA, Docosahexaenoic acid; HDL-C, HDL cholesterol; HDL-D, Mean diameter for HDL particles; IDL-C, Cholesterol in IDL; IDL-L, Total lipids in IDL; LDL-C, LDL cholesterol; LDL-D, Mean diameter for LDL particles; L-HDL-C, Cholesterol in large HDL; L-HDL-L, Total lipids in large HDL; L-LDL-L, Total lipids in large LDL; L-VLDL-L, Total lipids in large VLDL; M-HDL-C, Cholesterol in medium HDL; M-HDL-L, Total lipids in medium HDL; M-LDL-L, Total lipids in medium LDL; MUFA, Monounsaturated fatty acids; MUFA/FA, Ratio of monounsaturated fatty acids to total fatty acids; M-VLDL-L, Total lipids in medium VLDL; nm, nanometer; Omega-3/FA, Ratio of omega-3 fatty acids to total fatty acids; Omega-6/FA, Ratio of omega-6 fatty acids to total fatty acids; PUFA, Polyunsaturated fatty acids; PUFA/FA, Ratio of polyunsaturated fatty acids to total fatty acids; SFA/FA, Ratio of saturated fatty acids to total fatty acids; S-HDL-C, Cholesterol in small HDL; S-HDL-L, Total lipids in small HDL; S-LDL-L, Total lipids in small LDL; S-VLDL-L, Total lipids in small VLDL; VLDL-C, VLDL cholesterol; VLDL-D, Mean diameter for VLDL particles; XL-HDL-C, Cholesterol in very large HDL; XL-HDL-L, Total lipids in very large HDL; XL-VLDL-L, Total lipids in very large VLDL; XS-VLDL-L, Total lipids in very small VLDL; XXL-VLDL-L, Total lipids in chylomicrons and extremely large VLDL.*

*Concentrations are presented in mmol/l unless stated otherwise.*
*Mean (SE mean), median (IQR). Independent samples T-test or Mann-Whitney U test*
*Missing data: Alive: Creatinine n=1. Dead: Creatinine n=1, Phenylalanine n=1.*

**Supplementary figure 1. Correlation between albumin measured by the Nightingale platform and LUMC laboratory.**

**
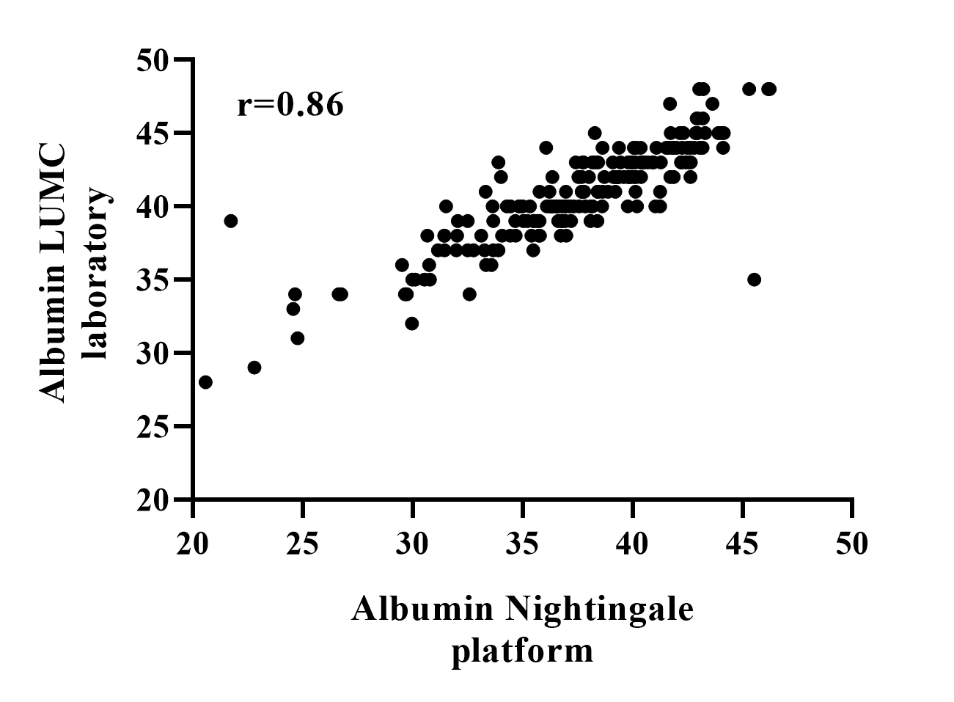
**

Abbreviation: LUMC, Leiden University Medical Center.

Pearson correlation between albumin measured by the Nightingale platform and serum albumin measured in the LUMC laboratory.
